# Forest Roles in Particle Removal during Spring Dust Storms on Transport Path

**DOI:** 10.3390/ijerph17020478

**Published:** 2020-01-11

**Authors:** Wenjun Wei, Bing Wang, Xiang Niu

**Affiliations:** 1 Research Institute of Forest Ecology, Environment and Protection, Chinese Academy of Forestry, Beijing 100091, China; wwj0318@126.com (W.W.); wangbingcfern@163.com (B.W.); 2 Liaoning Academy of Forestry, Shenyang 110032, China; 3 Key Laboratory of Forest Ecology and Environment of National Forestry and Grassland Administration, Beijing 100091, China; 4 Dagangshan National Key Field Observation and Research Station for Forest Ecosystem, Xinyu 338033, China

**Keywords:** forests, particles removal, dust storm, dust transport path

## Abstract

The transpacific transport of mineral dust often occurs in spring, and a large amount of aeolian dust is deposited in the Asian continent and north Pacific. Moreover, a heterogeneous reaction occurs when dust particles are mixed with man-made pollution gases and particles. In the present study, atmospheric PM_10_ and PM_2.5_ concentrations were investigated, and a scanning electron microscope and an X-ray energy spectrometer were used to analyze the effects of dust resistance and capture by forests. It showed that (1) the PM_2.5_ and PM_10_ concentrations during a dust storm, on sunny days, and during light pollution periods, were higher in the non-forest covered area (NFC area) than in the forest covered area (FC area), except during heavy pollution events; which suggests that the forests have a strong effect on dust resistance; (2) the PM reduction efficiency of forests was highest on sunny days, followed by light pollution periods, heavy pollution periods, and during the dust storm; (3) after the dust storm, TSP captured by leaves significantly increased, especially for the broadleaved tree species; and the particulates number in the grooves on leaves’ surface increased particularly sharply. This study will help improve the dust resistance and retention efficiency of forest shelterbelt projects during dust storms.

## 1. Introduction

The annual dust emissions in East Asia account for approximately 25% of the total global dust emissions [1]. These large emissions have a significant influence on the global radiation balance, climate, ambient air quality, and human health [2,3,4]. The trans-Pacific transport of mineral dust from East Asia to North America frequently occurs during springtime [3,5,6,7]. During spring, the Gobi region is affected by the Mongolian cyclone, which is the main factor behind the severe Asian dust storms [8]. The long-distance transport of dust plumes can alter the atmospheric conditions on regional and global scales [9,10]. In the transmission process, dust particles can mix with a variety of man-made pollution gases and particle emissions [11]. Many heterogeneous reactions can occur on the surface of dust particles, and the moisture absorption ability of ageing dust particles is enhanced [12,13]. It is possible to activate dust particles in the transport process, which then form cloud condensation nuclei, and change the physical and chemical properties of aerosols [14]. A dust emission event occurred between 3 May and 8 May, 2017. It was found to originate from the deserts of Central and East Asia (including the Mongolian Gobi Desert, the Taklimakan Desert, and the Alxa Desert) [15,16], where many dust events occur. The Alxa Desert in western Inner Mongolia and the Gobi Desert of Mongolia were found to be the main dust sources during this dust storm event, and the degraded air quality could have affected more than 700 million Chinese people living in the path of the dust storm [15]. Simulations by the Weather Research and Forecasting with Chemistry model indicated approximately 29.7 Tg of dust was emitted from dust sources in Mongolia and China, subsequently, 20.4 and 5.3 Tg of aeolian dust was deposited across continental Asia and the North Pacific Ocean, respectively [15]. The dust storm transported north-eastward to southern and eastern Russia and the Bering Sea, eastward to the Korean Peninsula and Japan, and southward to south-central China [16].

Vegetation can effectively improve air quality by reducing the pollution caused by atmospheric particulate matter (PM) through dust resistance, dust retention, and dust reduction [17,18,19,20,21,22,23]. It has been shown that increasing the surface coverage, leaf area, and the vegetation community vertical levels can enhance the ability of vegetation to reduce PM [24,25,26,27]. The resistance effects of vegetation on PM are not only related to leaf surface properties, canopy morphology, structure configuration, branch and leaf density, leaf surface inclination, and other factors, but are also affected by natural environmental factors, such as precipitation, strong wind, and dust storms, and human factors, such as traffic flow and heating [18,25,28,29,30]. Vegetation resistance has a positive effect on the removal of PM. Previous studies have mainly focused on the extent to which vegetation can reduce PM levels, and the processes and mechanisms underlying this reduction [31,32,33,34,35,36,37].

East Asian dust storm-prone areas are mainly in the 35–45° N region, and Beijing is just downwind of this area with a population of 21 million [38]. Furthermore, it is an important channel for dust aerosols that can transport dust across Asia in a downwards direction. Therefore, it is important to study dust aerosols in Beijing. This study compared the variations in atmospheric PM_10_ and PM_2.5_ concentrations between the dust storm occurrence period (dust storm period) and the non-dust storm period (non-dust storm period). Factors, such as sunny days, and light and heavy pollution periods, were investigated in a forest-covered area and an adjacent non-forest-covered area to assess whether the resistance effect of forests on different sized particles is the same during the dust and non-dust periods, and to determine the extent of the forest resistance effect. The increase in the dust retention capacity of the leaves from different tree species before and after dust storms and how the microstructure of tree leaves affects the increase in dust capture capacity were also determined. The changes to the element composition of the particles on the leaves before and after the dust storm and the source of the particles during the two periods were also investigated. The purpose of this study was to provide a scientific basis for the restoration and reconstruction of vegetation in dust source areas and to suggest ways of alleviating PM pollution caused by dust storms. This study provides a database that can be used to improve the dust resistance and dust retention efficiency of forest shelterbelt projects during the dust storms that occur on dust transport paths across the world.

## 2. Data and Methods

### 2.1. Air PM Concentrations and Meteorological Data

**Figure 1:**
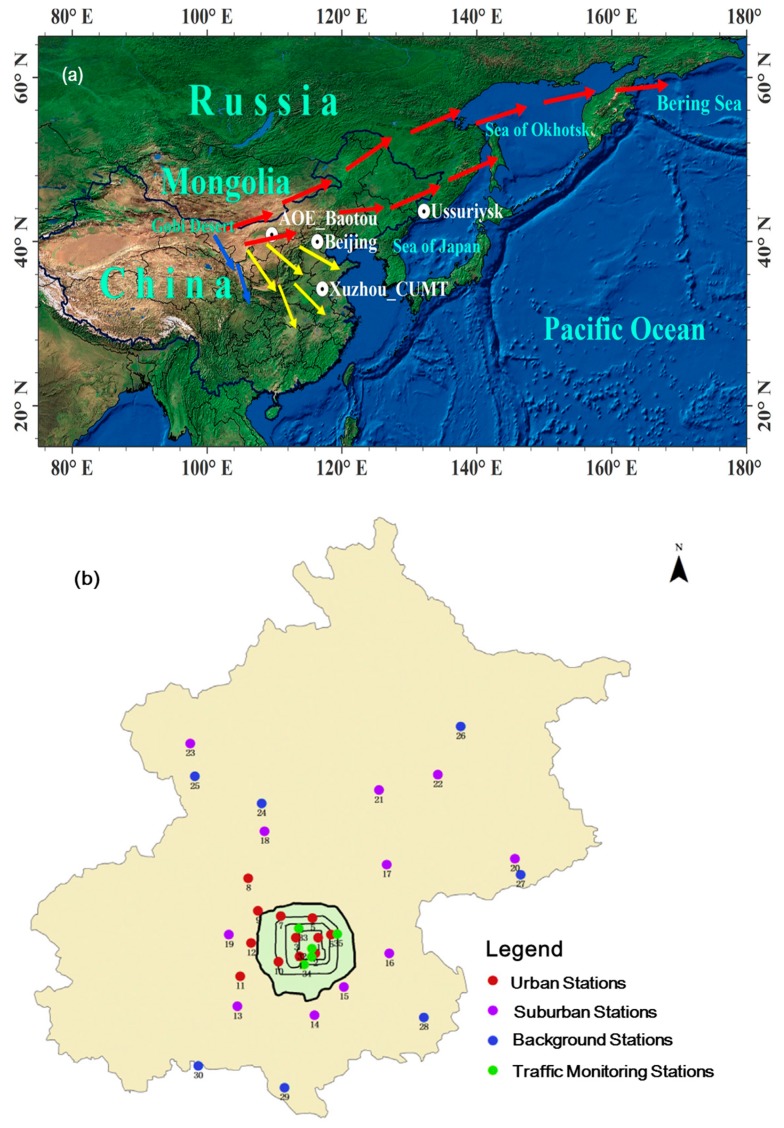
Transport path for dust event on May 2017 (**a**). The arrows show the dust transport directions, red arrows denote north-eastward transport, yellow arrows denote eastward transport, and blue arrows denote southward transport [16]. Location of air quality monitoring stations (**b**). Northern New Area stations is 8, and 9 is Beijing Botanical Garden station in Figure [39].

**Figure 2:**
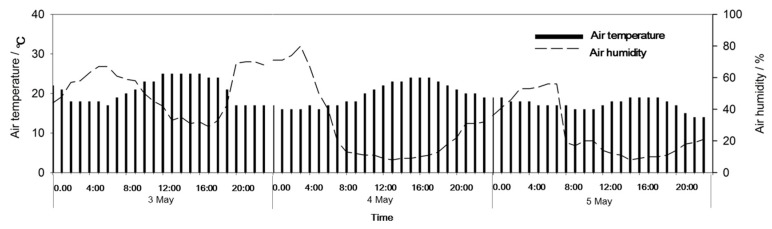
Air temperature and humidity during dust storm on 3–5 May, 2017 in Beijing.

**Figure 3:**
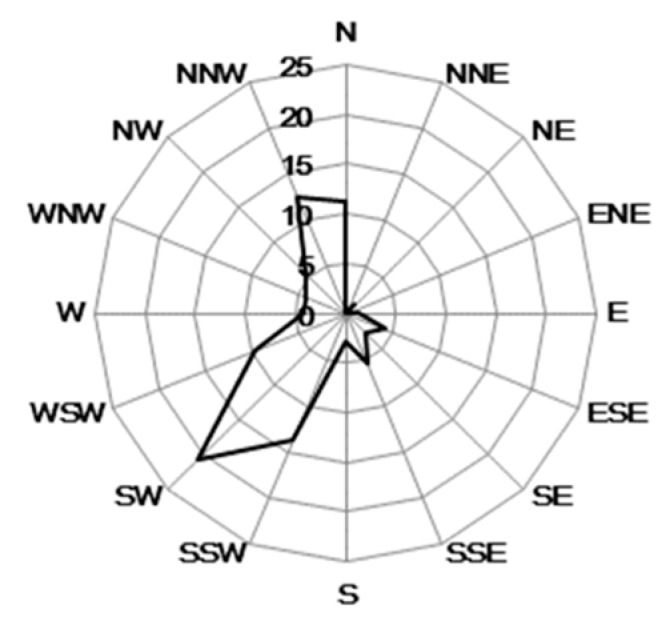
Frequency of wind direction during dust storm on 3–5 May, 2017 in Beijing.

The real-time monitoring data for PM_2.5_ and PM_10_ concentrations at 35 monitoring stations in Beijing have been published since January 2013 (http://zx.bjmemc.com.cn/) [39]. The locations of these 35 monitoring stations are shown in Figure 1 [39]. Two monitoring sites, Beijing Botanical Garden (forest-covered area, FC area, 9 in the Figure 1) and New Northern Haidian area (no forest cover area, NFC area, 8 in the Figure 1), were selected for this study. The atmospheric PM_10_ and PM_2.5_ concentrations in the FC area and the NFC area were compared during the dust storm from 3 May to 5 May, 2017. In order to better illustrate the dust removal efficiency of forests during dust storms, a comparative study, based on the weather conditions and the real atmospheric particulate pollution levels, was conducted between 16 April and 19 April (heavy pollution event), 6–8 May (sunny days without pollution event), and 16–21 May (light pollution event). In April and May, deciduous tree species in Beijing are in their leaf expansion period. Therefore, the forest growth conditions can be considered to be the same. The historical data provided for the websites has not been officially downloaded, and we used webserver for automatically downloading 1 h intervals real-time data from January to June 2017. Some transfer mistakes occurred during the data collection period, which meant that some of the data were missing. Meteorological data, mainly wind data, were obtained from the real-time weather collection data set for Haidian district and from the China Weather Network (www.weather.com.cn) (Figure 2, Figure 3 and Figure 5). Approximately 41 days was the rainfall-free period before the dust storm. The last rain fall before the dust storm was on 23 March, 2019, and precipitation was merely 2 mm.

The PM_2.5_ and PM_10_ reduction efficiencies were used to evaluate the dust resistance effect of forests [27,35]. The particle removal efficiency shows the differences between the FC area and the NFC area, and was calculated using Equation (1):

(1) $$E=\frac{C_{NFC}-C_{FC}}{C_{NFC}}\times 100\%$$

where *C_NFC_* denotes the particle concentration for the NFC area (μg m^−3^) and *C_FC_* denotes the particle concentration for the FC area (μg m^−3^).

### 2.2. Determination of Leaf Area

A scanner (Canon LIDE 110, Canon, Tokyo, Japan) was used to scan the leaves of broad-leaved tree, and the images were turned into a black and white image. Then, Adobe Photoshop (Photoshop CS2, Adobe, San Jose, CA, USA) was applied to obtain the black area, which represents the leaf surface area of the broad-leaved trees.

**Figure 4:**
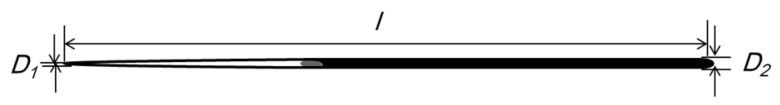
A sketch of the diameter at the upper and lower tip (*D_1_ and D_2_*), and length of the needle(*l*).

Around 100 g of needles from *Pinus tabulaefolius* or *Cedrus deodara* were sampled, and their diameters and lengths were measured. After needles were assumed as truncated cones, their average surface area was calculated by using Equation (2) [25]:

(2) $$A_{needle}=\frac{1}{2}\pi \cdot \left(D_{1}+D_{2}\right)\cdot {\left[\frac{1}{4}\cdot {\left(D_{2}-D_{1}\right)}^{2}+l^{2}\right]}^{\frac{1}{2}}$$

where *D_1_* is the average needle diameter at the upper tip; *D_2_* is the average needle diameter at the lower tip; and *l* is the average needle length (Figure 4). The total needles surface area was calculated through multiplying the needles number by the average needles surface area.

### 2.3. Leaves Dust Adsorption Abilities

Three evergreen tree species (*Platycladus orientalis*, *P. tabulaefolius*, *C. deodara*) and three deciduous tree species (*Ginkgo biloba*, *Acer truncatum*, and *Populus tomentosa*) leaves were collected from the FC area on 2 May (before dust storm), when it was known that the dust storm was going to start the next day through weather forecast, and on 6 May (after dust storm), 2017, when the dust storm ended. Overhead shears were used to collect about 100 g of leaves from each tree. The leaves were collected from east, west, north, and south parts of the canopy, and from three positions (top, middle, and bottom) in the canopy. The perennial leaves were collected from all three evergreen species (*P. orientalis*, *P. tabulaefolius*, and *C. deodara*). The leaves were complete, and free from disease and insect pests. Three trees with good growth statuses and mean diameters at breast height (DBH) were selected for each species as duplicates. The collected fresh leaves were put into clean, anti-static, self-sealing bags and brought back to the laboratory for using.

**Figure 5:**
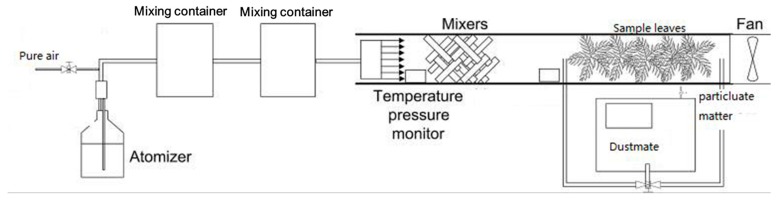
A sketch of the wind tunnel experimental setup [41].

The fine particles on the leaves were disturbed and mixed to form aerosols according to the principles behind wind erosion [40,41] (Figure 5). This process was replicated three times for each tree species. The particle absorption amount of leaves was measured in a wind tunnel that was 0.5 m wide, 0.5 m high, and 1 m in length [41]. Some leaves for measurement were cut freshly. To make sure that most airflow went through the leaves, they were tiled and stacked evenly. Leaves and branches occupy a total length of 1 m in the wind tunnel. The experiment was carried out at a wind speed of 20 m s^−1^, which meant that each leaf potential was able to be measured by dividing the total particles quantities captured by the leaves number.

The filtered indoor air with fairly constant concentration (<1 μg m^−3^) was sampled by wind tunnel, and the total volume is about 10 m^3^. Firstly, the leaves were put in the tunnel, and pure air without any PM was introduced into the tunnel through a plenum with multiple openings. Secondly, a fan which generated a wind speed of 20 m s^−1^ was switched on to blow through the leaves in the tunnel, and it lasted for 6–10 min to ensure that all PM on the leaves surface was suspended in wind tunnel [29]. Finally, a DustMate environmental monitor (DustMate, Turnkey, Cheshire, UK) was applied to determine the particulate concentration in the tunnel air [42,43]. Equation (3) was used for calculating the captured PM amount per unit of leaf area for the different tree species [30]:

(3) $$M_{i}=\overset{n}{\underset{1}{\sum }}m_{ij}/S_{i}$$

where *M* represents the leaves surface absorbed PM of different tree species (g cm^−2^), *i* represents the different tree species, *j* represents the particulate sizes, *n* represents three replicates, *S* represents the leaf surface areas (cm^2^), and *m_ij_* represents the total suspended particulate matter (TSP), PM_10_, and PM_2.5_ (μg) amount.

### 2.4. Leaf Microstructure Observation, Leaf Surface Particle Morphology, and Element Composition Analysis

Small cubes (4 × 4 mm) from the middle of the fresh leaves were cut and fixed in 2.5% (volume fraction) glutaraldehyde solution. Then ethanol was added to dewater the leaves, and the samples were sprayed with a conductive coating. The surface microstructure and particle morphology of the leaves were observed and analyzed by a scanning electron microscope (SEM) (S-3400, Hitachi, Tokyo, Japan) and an X-ray energy spectrometer (EDS) (550i, IXRF, Texas, USA). The images were taken at 500×, 1000× or 8000× resolution. The leaf surface microstructure images were at 500× resolution, the particle morphology images were at 1000× resolution, and the X-ray energy spectrum composition analysis of the particles used an 8000× resolution. SEM-EDS can be used to observe the particles on the leaf surface, and its energy spectrum electron probe can be used to analyze the particles, which meant that the energy spectrum and element content of the particles could be obtained. The development time for *G. biloba* leaves was relatively short. Therefore, the particles on the leaf surface of *G. biloba* were mainly from the dust storm period. *P. orientalis* and *P. tabulaefolius* are evergreen species, which meant that they could be used to analyze the source of the PM. The PM on *P. tabulaefolius* was greater than on *P. orientalis* before the dust storm, and the PM >10 μm was also greater. Therefore, *P. tabulaefolius* was used to compare the differences in element compositions of the PM before and after the dust storm and to determine the source of the PM.

## 3. Results

### 3.1. Dust Resistance Effect of Forests during Dust Storms

**Figure 6:**
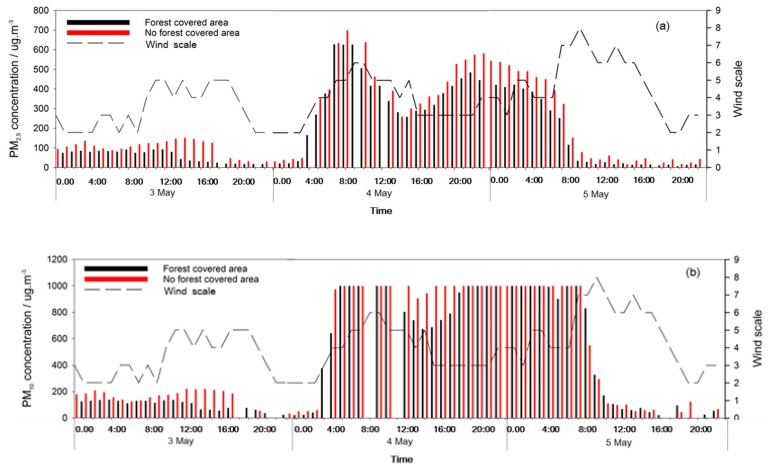
PM_2.5_ (**a**) and PM_10_ (**b**) concentrations changes during dust storm.

The PM_10_ and PM_2.5_ concentrations in the NFC area were a little higher than in the FC area during the dust storm (Figure 5). When the wind speed reached peak, the PM_10_ concentrations in both NFC and FC areas rapidly exceeded 1000 μg m^−3^ (Figure 6b). However, the PM_2.5_ concentration gradually rose and peaked (Figure 6a). At the end of the dust storm, due to the decrease in the transport of exogenous particles, the PM_10_ and PM_2.5_ concentrations sharply decreased. Moreover, as the wind speed peaked, there were little differences for the PM_10_ and PM_2.5_ concentrations between the NFC and FC areas.

### 3.2. Dust Resistance Effect of Forests during Non-Dust Storms Periods

**Figure 7:**
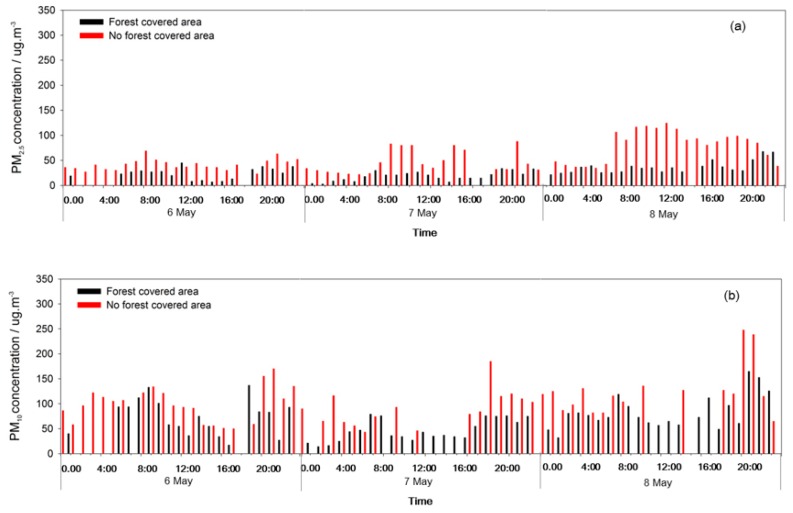
PM_2.5_ (**a**) and PM_10_ (**b**) concentrations changes in sunny days.

**Figure 8:**
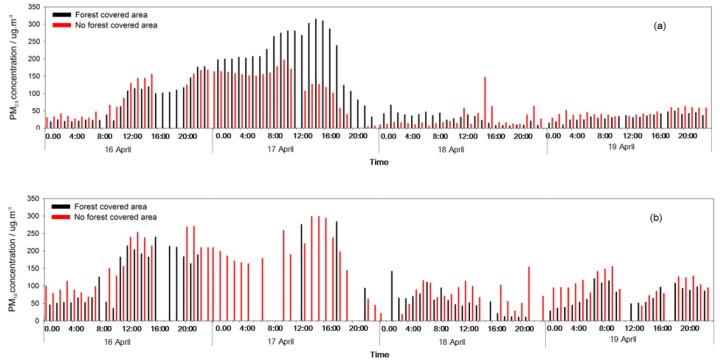
PM_2.5_ (**a**) and PM_10_ (**b**) concentrations changes in light pollution event.

**Figure 9:**
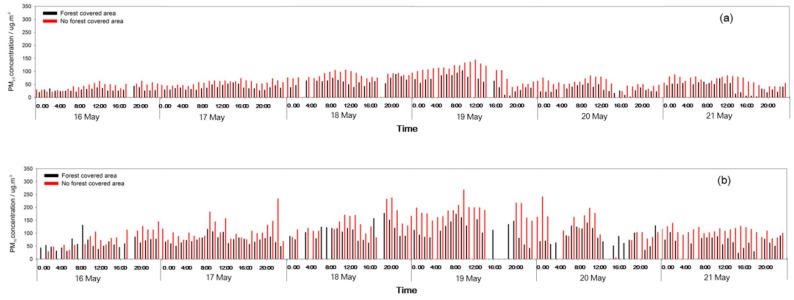
PM_2.5_ (**a**) and PM_10_ (**b**) concentrations changes in heavy pollution event.

During sunny days and the heavy pollution period, the PM_10_ and PM_2.5_ concentrations were obviously lower in the FC area than in the NFC area. The forests had a strong dust resistance effect, and the dust resistance towards PM_10_ was stronger than for PM_2.5_. During the light pollution period, the PM_10_ concentration was lower in the FC area than in the NFC area. However, the PM_2.5_ concentration was particularly higher in the FC area than in the NFC area on 17 April (Figure 7, Figure 8 and Figure 9).

**Figure 10:**
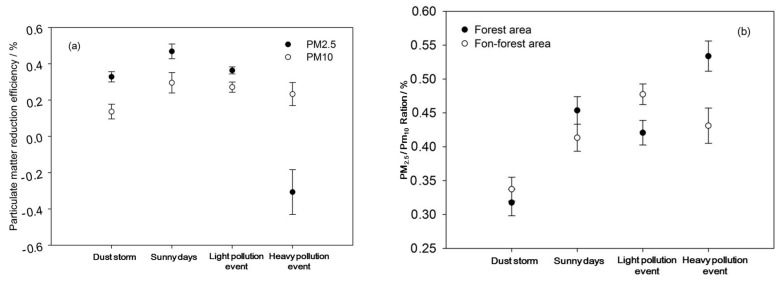
Reduction efficiency for PM_2.5_ and PM_10_ (**a**) and PM_2.5_/PM_10_ ratio (**b**) in forest area and non-forest area.

The overall reduction efficiency for PM_2.5_ was higher than for PM_10_, except for during a heavy pollution event. It can be seen that forests have a prominent effect on the reduction of fine particles, such as PM_2.5_. The reduction efficiency results for PM_10_ under the different weather conditions showed that forests were most efficient on sunny days, followed by the light pollution event, then the heavy pollution event, and the dust storm period was the least efficient one; however, for PM_2.5_, the reduction efficiency of the heavy pollution event was the least (Figure 10a).

Overall, there was little difference in the PM_2.5_/PM_10_ ratios between the NFC area and the FC area. The PM_2.5_/PM_10_ ratios were lowest during the dust storm, and PM_10_ was the main component of the dust storm. The PM_2.5_/PM_10_ levels on sunny days and during the light and heavy pollution periods showed no obvious trend. The particulate concentration differences between the three weather conditions were small, but they were all higher than during the dust storm (Figure 10b).

### 3.3. Forest Dust Capture Ability during Dust Storms

**Figure 11:**
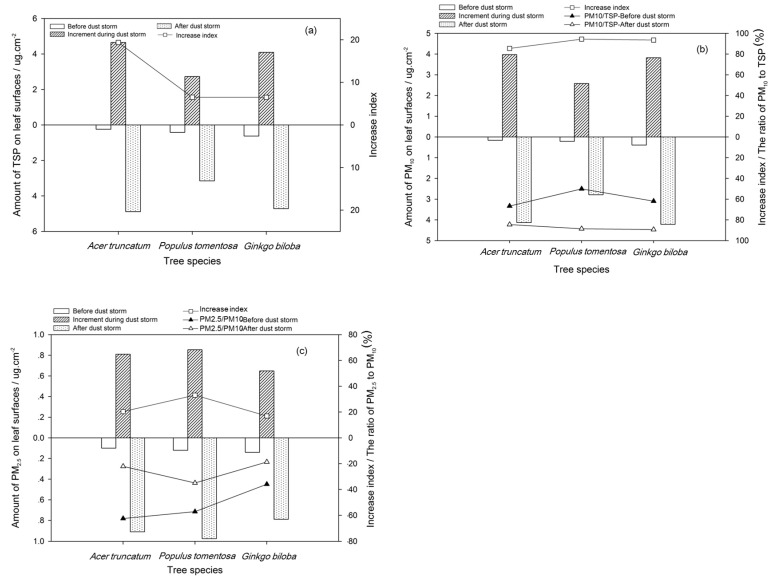
Total suspended particulate matter (TSP) (**a**), PM_10_ (**b**) and PM_2.5_ (**c**) on the leaf surfaces of the deciduous tree species before and after dust storm and its increase index.

**Figure 12:**
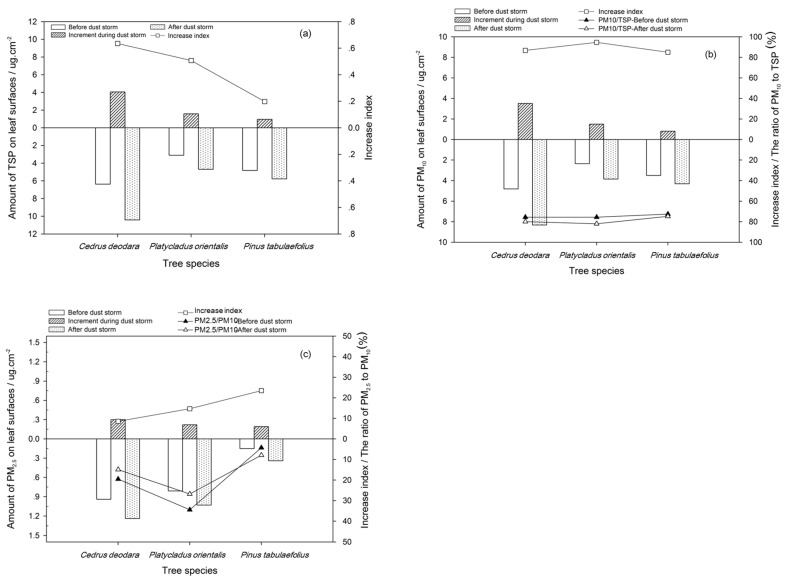
TSP (**a**), PM_10_ (**b**), and PM_2.5_ (**c**) on the leaf surfaces of the evergreen tree species before and after dust storm and its increase index.

After the dust storm, the total PM amount accumulated on the leaves significantly increased. The TSP increase for the three kinds of broad-leaved tree species (deciduous tree species) was, from large to small, *A. truncatum* (4.65 μg m^−^^2^), *G. biloba* (4.01 μg m^−^^2^), and *P. tomentos* (2.73 μg m^−^^2^). The TSP for these trees was 649%–1937% more after the dust storm than before the dust storm, especially there accumulated more PM_10_ on leaves (Figure 11). The TSP order for the three conifer species (evergreen species), from large to small, was *C. deodara* (4.05 μg m^−^^2^), *P. orientalis* (1.58 μg m^−^^2^), and *P. tabulaefolius* (0.95 μg m^−^^2^) (Figure 12). The increase in TSP adsorption by the three conifer tree species was 19.63% to 63.55% before the dust storm (Figure 12a).

The quantity of PM_10_ deposited on the leaves was higher than the amount of PM_2.5_, which was directly related to the fact that the PM_10_ concentration in the air was much higher than the PM_2.5_ concentration during the dust storms. The PM_10_ accounted for 84.99%–94.59% of the increase in total PM on the leaves after the dust storm, whereas PM_2.5_ only accounted for 8.54%–33.11%.

### 3.4. Effects of Leaf Microstructure on Dust Retention

**Figure 13:**
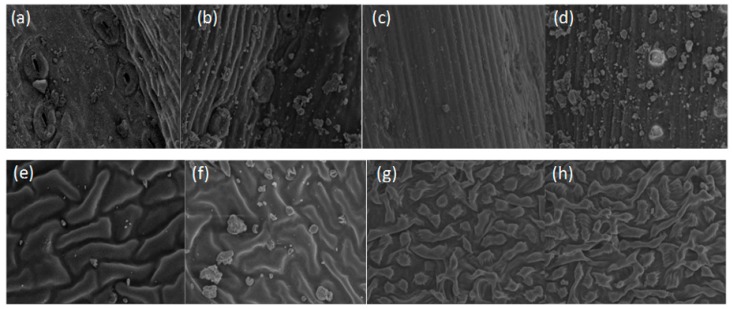
Scanning electron micrographs of leaf surfaces before and after dust storm. *Platycladus orientalis*, (**a**) before dust storm, (**b**) after dust storm. *Pinus tabulaefolius*, (**c**) before dust storm, (**d**) after dust storm. *Ginkgo biloba*, adaxial surface, (**e**) before dust storm, (**f**) after dust storm; abaxial surface, (**g**) before dust storm, (**h**) after dust storm.

The PM numbers on the leaves of the *P. orientalis*, *P. tabulaefolius*, and *G. biloba* significantly increased after the dust storm (Figure 13). The large-particle-sized dust was significantly greater than the smaller-particle-sized dust. In particular, the number of particulates in the grooves on the adaxial surface of the *G. biloba* leaves increased sharply, and the number of fine particulates in the grooves of the *P. orientalis* leaves also significantly increased (Figure 12a and Figure 13b,e,f). The increase in the fine particulate levels on the leaf surfaces of the *P. tabulaefolius* leaves was slightly lower (Figure 13c,d). This was consistent with the results that showed that there has been a continual increase in PM retention on these tree species. There was no significant increase in the amount of PM on the abaxial side of the *G. biloba* leaves (Figure 13g,h).

The *P. orientalis* and *P. tabulaefolius* leaves, and the adaxial surface of the *G. biloba* leaves had clearly retained dust after the dust storm, and they were uniformly covered with different sized particles. However, the particle distribution on these leaves before the dust storm was more spatially heterogeneous and irregular.

### 3.5. Differences in Particle Morphology and Element Composition Before and After the Dust Storm

**Figure 14:**
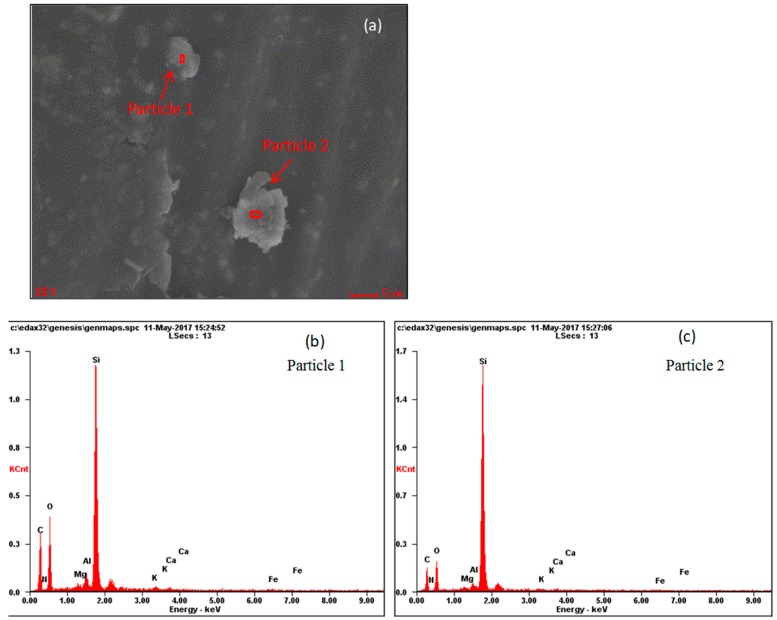
SEM micrographs of *Pinus tabulaeformis* leaves before dust storm (**a**), and EDS pictures of two PM_10_ particles (**b**,**c**) (×8000).

**Figure 15:**
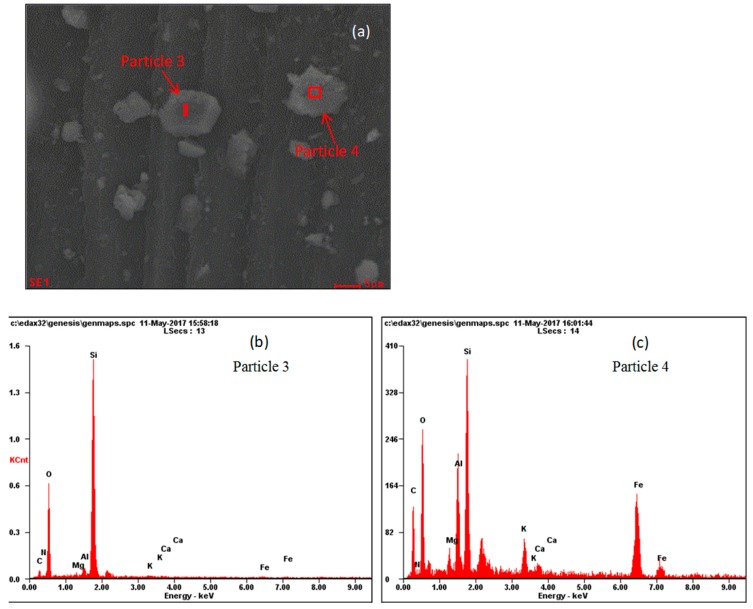
SEM micrographs of *Pinus tabulaeformis* leaves after dust storm (**a**), and EDS pictures of two PM_10_ particles (**b**,**c**) (×8 000).

Figure 14a and Figure 15a are leaf surface SEM images of *P. tabulaefolius* before and after the dust storm, and Figure 14b,c and Figure 15b,c show the EDS energy spectra for the elemental analysis of two PM_10_ particles on the leaf surface. Table 1 shows the element contents of four PM_10_ particles—two particles from leaves collected before the dust storm and two particles from leaves collected after the dust storm. The main elements in the four PM_10_ particles were silicon (Si), oxygen (O), and aluminum (Al). The four PM_10_ particles were all sialate particles.

## 4. Discussion

### 4.1. Dust Resistance Role Played by Forests during Dust Storms

Forests have a strong effect on dust resistance during the early stages of a dust storm. However, due to the considerable wind speed and high concentrations of exogenous dust during the dust storm, the dust resistance effect of forests is very weak during the outbreak of a dust storm. The atmospheric PM_10_ and PM_2.5_ concentrations in the NFC areas were higher than in the FC areas. At the end of the dust storm, the decrease in the quantity of exogenous dust and the increase in the wind speed meant that the PM_10_ and PM_2.5_ concentrations in the FC and NFC areas decreased sharply. Furthermore, the wind played a strong role in the diffusion of particulate pollution. In terms of PM reduction efficiency, forests on sunny days have the highest reduction efficiency, followed by the light pollution and heavy pollution periods, and dust storms. Therefore, forests can effectively reduce the PM_10_ and PM_2.5_ concentrations in the air during sunny and light pollution periods. During heavy pollution periods, PM_2.5_ reduction efficiency can have negative values in the FC areas. This is what mainly happens when heavy pollution occurs during the flowering season of some plants in Beijing Botanical Garden (FC areas), such as *Amygdalus triloba*, *Amygdalus persica* L. var., and *Prunus persicaf*. The large amount of pollen and biogenic volatile organic compounds (BVOCs) discharged during flowering directly increased the primary PM levels and generated secondary organic aerosols, which is important because BVOCs are precursors in a series of oxidation and gas/particle distribution processes that occur in the atmosphere [32,44,45,46,47]. Therefore, the pollution due to fine particulates rises. Furthermore, studies have shown that the higher the concentration of BVOCs released by plants, such as monoterpenes, the higher the concentration of secondary organic aerosols generated by photooxidation, and, under certain environmental conditions, the higher the concentration of PM formed by nucleation. These factors lead to an environmental increase in PM_2.5_ concentration [48,49,50].

In this study, the variation trend for PM_10_ and PM_2.5_ concentration in the dust storm during May 2017 in the NFC area was basically consistent with the trends reported by Zhuang et al. [38]. It was also very close to the Chengde station results reported by She et al. [16]. Furthermore, the peak concentration for PM_10_ also exceeded 1000 ug m^−3^ [15,38], and the peak time was also similar to the Chengde station results [16].

The overall reduction efficiency for PM_2.5_ was higher than for PM_10_. It can be seen that forests have a prominent effect on fine PM reduction, which is mainly because tree leaves can improve air quality by capturing PM. It has been shown that the tree PM capture efficiency mainly depends on wind speed, particle size, and the physical and biological characteristics of the tree canopy [28]. The mechanism underlying the capture of fine PM by leaves depends on the size of the PM and its floating speed. The deposition of particles smaller than 0.5 microns mainly depends on the Brownian diffusion principle, whereas the deposition of particles larger than 0.5 microns mainly depends on retardation. The leaf microstructure has a considerable influence on PM deposition. It can be used to explain the effect of wind speed on the capture of different sized particles [51]. When air flow pushes PM_2.5_ against the tree, it gets intercepted by the roughness and wetness of its leaves and bark, and the particles are fixed by the rough epidermis, cuticle, trichomes, gullies, and other leaf microstructures. Furthermore, special secretions on the leaf surface embed or stick PM_2.5_ to the leaf surface [17]. These secretions capture some of the PM_2.5_ in the air and retain them on the plant surface, which effectively reduces the PM_2.5_ concentration in the air. The PM_2.5_/PM_10_ ratio is the smallest component in dust storms, and PM_10_ is the largest particle component in dust storms, but its levels are far lower in dust storms than in non-dust storms [38]. The PM_2.5_/PM_10_ ratios during sunny, and light and heavy pollution periods do not seem to have obvious variation trends and the differences between the periods are small. However, the levels are higher than during a dust storm, which indicates that the proportion of coarse particles during dust storms significantly increases [52]. When there was a heavy pollution event, the PM_2.5_/PM_10_ ratio in the FC area was much higher than in the NFC area, which was mainly due to the pollen and BVOCs emissions from trees that are in flower during heavy hazy weather. These promote the generation of secondary derivative particles and aggravate the fine particulate pollution in the vegetation area [48]. In addition, the nitrogen oxidation ratio and the sulfur oxidation ratio were much greater during hazy days than during non-hazy days. This implies that the formation of sulfate and nitrate was greatly accelerated due to the heterogeneous or multiphase reactions of NO_2_ and SO_2_ with PM_2.5_ [35]. The cause of the rise in the PM_2.5_/PM_10_ ratio needs to be explored further. The results showed that temperature was negatively correlated with PM_2.5_ and that relative humidity was positively associated with PM_2.5_, which was consistent with the results of this study [23].

### 4.2. Effect of Tree Leaves on Dust Retention during a Dust Storm and Its Influencing Factors

After the dust storm, the increase in fine PM on the leaf surfaces of coniferous trees (*Platycladus orientalis*, *P. tabulaefolius*, and *C. deodara*) was smaller than on broad-leaved trees (*A. truncatum*, *P. tomentosa*, and *G. biloba*). The broad-leaved species were all deciduous species. In April, the deciduous species in Beijing begin to germinate and spread their leaves, which means that the PM on new leaves was low in May. However, the conifers are all evergreen trees, and the PM levels on their leaf surfaces are high in May. In addition, the amount of PM that accumulated on the surface of the leaves was close to saturation because none of the PM was washed off due to the lack of rainfall before May. Therefore, the increase in PM accumulation on the leaves during the dust storm was far less than on deciduous tree species. Although the three broad-leaved tree species showed a large increase in the amount of deposited PM, the amount of accumulated PM after the dust storm was lower than that of the three coniferous tree species, which was consistent with the results from previous studies [51].

In general, the PM_10_ amounts on the leaf surfaces of the six tree species were higher than the PM_2.5_ levels. Similarly, the increase in the amount of PM_10_ captured by the leaves the after the dust storm was greater than the increase in the amount of captured PM_2.5_. The reason is that the PM_10_ concentration in the air is much higher than the PM_2.5_ concentration during a dust storm. This is because there are more PM_10_ sources that can contaminate the air [16]. The number of particles in the grooves on the adaxial surface of the *G. biloba* leaves increased sharply, and the number of fine particles in the grooves of *P. orientalis* also significantly increased. The grooves on the leaf surface play a more significant role in capturing air particles when particle matter capture increases sharply. However, it has been shown that the fine particles on the leaf surface that have a large diameter are unstable and can be easily eluted by external forces (rain, wind, etc.) [18]. This means that leaves can repeatedly capture the PM in the air and this improves their ability to ecologically purify the air of PM.

Approximate 2.85 × 10^7^ t PM_2.5_ was removed from the atmosphere for the Three Northern Shelter Forest during 1999 and 2010 [53]. It is projected to continue till 2050, and there will be more than 3 million hectares of trees planted every year [54], which will enhance continuously the PM_2.5_ uptake by forest. Forest configuration, structure, height, and degree of forest belt fully should be paid more attention to ensure that the project will play an increasingly important role in the improvement of the regional-scale air quality in the coming years, especially tree species selection. It should be paid more attention on the selection of tree species with high particulate matter capture ability, meanwhile, fast-growing species should be planted with slow-growing species, and evergreen trees should be planted with deciduous trees, which will help improve the dust removal efficiency of forest shelterbelt projects effectively during dust storms.

The dust storms in spring, during the leaf expansion time for deciduous broadleaf tree species, are quite short. However, the potential amount of fine PM that could be deposited on leaves is considerable. The effect of dust purification is strongest during dust storms. The fine particles captured by conifer trees with leaves that have a grooved structure can easily fall off due to the action of external forces. The repeated dust retention characteristics of the grooved structure improve fine PM retention when a dust storm is combined with a severe air particle pollution event.

The known morphologies and energy spectra of typical particles show that the four PM_10_ particles are all sialate particles. The PM in Beijing Botanical Garden before and after the dust storm was composed of elements from the earth’s crust and natural sources of pollution. However, the impact of human pollution sources was very small. The single particle mass spectrogram analysis of the dust storms undertaken by Zhuang et al. [38] showed that the calcium, iron, aluminum, and silicate signals were enhanced in the fine PM produced on a dust day. The dust also contained more crust elements, such as silicate, sodium, and aluminum. However, dust that has been allowed to age contains more secondary inorganic ions, such as sulfate and ammonium nitrate.

The results produced by this study are similar to the results reported by Zhuang et al. [38], which showed that the dust levels (55.3%) were highest on dust days. However, the results from this study are different for non-dust days. In the Zhuang et al. [38] study, the secondary inorganic sources accounted for 24.6% of the dust particles, motor vehicle exhaust sources accounted for 21.1%, and other dust sources accounted for 13.8% [38]. The main reason for the different results is that the sampling place in this study was Beijing Botanical Garden, which is located in a northwest suburb of Beijing. Therefore, the surrounding pollution sources are mainly natural dust. However, Zhuang et al. [38] chose Da Yang fang, Anwai, Chaoyang district, which is mainly surrounded by traffic pollution sources. This study shows that the particle size of the dust source increased significantly during a dust storm, and that the source of these particles is mainly the long-distance transmission from a dust source area.

## 5. Conclusions

As simulated by the WRF-Chem model, approximately 25.7 Tg of dust was deposited over the Asia-Pacific region, and 4 Tg of dust was suspended in the atmosphere during dust storm in May, 2017 [15]. The significantly deteriorated air quality affected people living in the path of the dust storm. Forests can effectively remove particles through dust resistance, dust capture, and dust reduction. Dust resistance of forest during dust storms on transport path was analyzed through paired comparison analysis with non-forest area and forest area, and a scanning electron microscope and an X-ray energy spectrometer were used to analyze the effects of dust capture by forests leaves.

The PM_2.5_ and PM_10_ concentrations during a dust storm, on sunny days, and during heavy pollution periods were higher in the NFC area than in the FC area, except during light pollution events; which suggests that the forests have a strong effect on dust resistance. The reduction efficiency of larger PM for forests was highest on sunny days, followed by light pollution periods, heavy pollution periods, and during the dust storm. It indicated that the effect of forest on dust reduction and dust resistance was subtly small, which attributed to the fast wind speed during the dust storm. However, in late dust storm, the forest roles in dust removal became significant. During heavy pollution periods, PM_2.5_ reduction efficiency can have negative values in the FC areas. It confirmed that the higher the concentration of BVOCs released by plants, the higher the concentration of secondary organic aerosols generated under certain environmental conditions, which aggravated the fine particles pollution. After the dust storm, TSP captured by leaves significantly increased, especially for the broad-leaved tree species; and the particulates number in the grooves on leaves surface increased particularly sharply. Therefore, selection of tree species with high PM capture ability on leaves (Cypress with scaly leaves or evergreen conifer species capable of secreting turpentine, mixed deciduous broad-leaved tree species capable of spreading leaves in April, leaf surface with deep groove, large roughness, complex surface texture) will help improve the dust removal efficiency of forest shelterbelt projects during dust storms and should not be ignored.

## Figures and Tables

**Table 1:** Element content of four PM_10_ particles on *Pinus tabulaeformis* leaf surfaces before and after dust storm (%).

Elements	Before Dust Storm				After Dust Storm			
	Particle 1		Particle 2		Particle 3		Particle 4	
	Mass Percent	Atomic Percentage	Mass Percent	Atomic Percentage	Mass Percent	Atomic Percentage	Mass Percent	Atomic Percentage
***N***	0.73	0.78	0.96	1.1	1.85	2.38	1.92	2.5
***O***	25.55	24.09	19.88	19.93	44.12	49.53	25.46	28.94
***Mg***	0.32	0.2	0.21	0.14	0.27	0.2	1.3	0.98
***Al***	1.7	0.71	0.88	0.52	1.38	0.92	5.8	3.91
***Si***	20.71	11.13	32.38	18.48	34.52	22.07	11.61	7.52
***K***	0.48	0.19	0.26	0.11	0.42	0.19	2.49	1.16
***Ca***	0.32	0.12	0.42	0.17	0.34	0.15	0.9	0.41
***Fe***	0.81	0.22	0.49	0.14	0.88	0.28	18.42	6
